# Supplementary material for: Evaluation of a service introduced to support young people at risk of suicide using a cohort design, a mixed methods analysis and cost-benefit analysis
Source: PLOS Ment Health. 2024 Jun 10;1(1):e0000043. doi: 10.1371/journal.pmen.0000043 (PMC12798205; doi:10.1371/journal.pmen.0000043)
Supplement: S1 Text — Table A in S1 Text: Cost-benefit analysis at 9 and 12 months. Table B in S1 Text: General assumptions. Table C in S1 Text: Cost item data sources and assumptions. Table D in S1 Text: Benefit values–data sources and assumptions. (DOCX) [file pmen.0000043.s001.docx]

# S1: Cost-benefit analysis

An indicative cost-benefit analysis was conducted for the 17 month evaluation period (February 2022 to June 2023) of the service. Costing data was provided by the service, and costs and benefits were calculated using publicly available data. Table S1 outlines the cost-benefits calculated for the 17-month program.

*Table A in S1 Text: Cost-benefit analysis*

| **Costs and benefit items** | **Benefits**  **9 months** | **Benefits**  **12 months** |
| --- | --- | --- |
|  |  |  |
| **Costs** |  |  |
| Staffing costs | $1,053,223 | $1,053,223 |
| Operating costs | $107,456 | $107,456 |
| Accommodation | $37,960 | $37,960 |
| Vehicle costs | $10,036 | $10,036 |
| **Total program costs** | **$1,208,674** | **$1,208,674** |
|  |  |  |
| **Patients (# admissions)** | 91 / (92) | 91 / (92) |
| **Cost per admission** | **$13,138** | **$13,138** |
|  |  |  |
| **Reduction in health service usage** |  |  |
| CATT outreach | $128,143 | $174,557 |
| Ambulance attended at home | $64,694 | $86,259 |
| Brought to ED by family/friends | $9,384 | $12,580 |
| Brought to ED by ambulance | $26,772 | $36,123 |
| Brought to ED by ambulance/police | $10,709 | $14,278 |
| Brought to ED by police | $10,709 | $16,063 |
| Attended ED – not admitted | $63,843 | $87,232 |
| Attended ED – admitted | $28,193 | $39,644 |
| Nights in hospital | $243,625 | $366,447 |
| **Total benefit related to reduction in health service usage** | **$586,072** | **$833,183** |
|  |  |  |
| **Increase in Quality of Life** | **$239,072** | **$306,447** |
|  |  |  |
| **Total program benefits** | **$825,144** | **$1,139,630** |
| **Benefits per admission** | **$8,969** | **$12,387** |
|  |  |  |
| **NET (COST) / BENEFITS** | **($383,530)** | **($69,044)** |
| **Net (cost) / benefits per admission** | **($4,169)** | **($751)** |
|  |  |  |

.

*Table B in S1 Text: General assumptions*

| **Cost element** | **Data sources / Assumptions** |
| --- | --- |
| Costing approach | Full (or absorption) costing approach used. This approach includes both variable (e.g., staffing) and fixed costs (e.g., vehicle/rent). |
| Admissions | Patient numbers (or admissions) provided by HOPE team for evaluation period February 2022 to June 2023. Included two re-admissions.  This represented the number of patients who completed the 12 week program. Patients not admitted to the program or who did not complete the program were not included in the admission numbers. |
| Program costs | Staffing numbers and rent were provided by the HOPE team.  Assumptions to calculate full program costs are detailed in Table 2. |
| Health service usage benefits | - Patient information for 70 admissions (69 patients) over 17 admitted to the HOPE program who were in the Alfred Hospital catchment area were used as the basis for calculating the reduction in health usage benefits for all benefits except emergency services attendance at home and friends or family bringing patient to ED. It was assumed that the remaining 22 admissions (19 under age of 17 and 2 referred by Monash Health) would have similar reductions in health usage. This information was sourced from Alfred hospital records. - For the remaining benefits (emergency attendance at home, and friends and family bringing patient to ED), the evaluation data for 10 patients over 17 was used as this information was not available in the Alfred hospital records. |
| QALY benefit calculated | Based upon QALY change at discharge from HOPE program and at three month follow-up. Applied as follows:   - Three months during admission. QALY improvement 0.290 which equated to monthly improvement of 0.0242 applied over three months - Nine months (or 6 months or 21 months) post discharge. QALY improvement 0.183 which equated to monthly improvement of 0.0153 applied over nine months |
| Time period | Costs were calculated for 17 months of the program.  Benefits were calculated as lasting for 12 months. Three months during admission, and 9 months post-admission based upon 3 and 6 month data collected.  Sensitivity analyses of benefits were calculated for nine months and two years. Nine months being the period for which the data was collected, and twenty-four months being period in which benefits may be maintained. |

*Table C in S1 Text: Cost item data sources and assumptions*

| **Cost element** | **Data sources / Assumptions** |
| --- | --- |
| Staffing costs | - Based on team of 6.8 FTE comprised of:   - Team Leader 0.3 FTE (Psychologist P4)   - Co-ordinator /Senior Clinician 1.0 FTE (Social worker SW3)   - Senior Clinician 0.80 FTE (RPN4)   - Youth worker 0.8 FTE   - Family peer worker 0.6 FTE   - Wellbeing worker 2.0 FTE   - Psychiatrist 0.2 FTE (Assumed only works at hospital/Year 4)   - Registrar 0.5 FTE (Assumed Year 4)   - Administrative support 0.4 FTE (Assumed Grade 2)   - Communications 0.2 FTE (Assumed Grade 3) - An additional worker on salary equivalent to Wellbeing worker also worked for 9 months and was included in staffing costs, but the position did not continue beyond Oct 2022 - Weekly salaries based upon 2022 year in EBA   - Psychologists = Medical Scientists, Pharmacists and Psychologists Victorian Public Sector 2021 - 2025   - Psychiatrists = Medical Specialists - VPS-Health Sector AMA Victoria ASMOF Single Interest Employers Enterprise agreement 2022-2026   - Registrars = Doctors in Training (Victorian Public Health Sector) (AMA Victoria/ASMOF) 2022-2026   - All other staff = Victorian Public Mental Health Enterprise Agreement 2021-2024   - Used highest level in each grade. For example, Wellbeing worker OT1, used level 7. - All staff work standard hours, no overtime or shift penalties - Training allowances for psychiatrist /registrar as per EBA - Qualification allowances included for three senior staff, team leader and senior clinicians (assumed masters for psychologist; post grad diploma for RPN4 and SW3) - Oncosts, say, 22.5%.   - Superannuation contributions 2022-23 year (10.5%)   - Workcover, payroll tax, recreation leave loading, long service leave provision (11.81%). Rates based upon Department of Treasury and Finance Budget guidance. Attachment E – Additional Guidance 2022-23 - No allowance made for recruitment costs, assumed part of the 11.81% as stated oncosts for workcover etc. only account for approx. 10% of oncosts. |
| Operating costs | - $13,747 per FTE - Allowance for stationery, telephone, printing, training and provision of a single PC - Department of Treasury and Finance Budget guidance. Attachment E – Additional Guidance 2022-23 |
| Rent | - Costs provided by Alfred - Separate building for the HOPE team, with annual rent 2022/23 |
| Vehicle costs | - Costs provided by Alfred - Assumed 1 pool care shared by the team - $590.30 per month - Includes lease costs, tolls and fuel costs |

*Table D in S1 Text: Benefit values – data sources and assumptions*

| **Cost element** | **Benefit** | **Data sources / Assumptions** |
| --- | --- | --- |
| Crisis and Assessment Treatment Team visit at home | $500 | - Cost developed based upon the following assumptions as no publicly available information - Assume 2 senior clinicians (use team leader hourly rate, plus oncosts, plus 15% allowance for shift penalties as after-hours service) - Cost per hour estimated to be $110 - Two hours to outreach, assess and write notes. Assume not admitted to hospital as this would increase hours required. - Total four hours @$110 = $440 plus allowance for vehicle. - Say $500 as similar to ambulance attendance but less expensive vehicle and equipment. |
| Ambulance treatment at home | $586 | - Based upon data collected from 10 people between Time 1 and Time 2 economic evaluation questionnaires. Assume Time 2 data remained same across remaining months. - Extrapolated across all 94 patients admitted to program - Cost assumed to be equivalent to fees charged $586 (2022 fee) - Sourced from https://www.health.vic.gov.au/patient-care/ambulance-fees |
| Ambulance – transported to hospital | $1358 | - Cost assumed to be equivalent to fees charged $1,358 (2022 fee) - Sourced from <https://www.health.vic.gov.au/patient-care/ambulance-fees> |
| Police required for transportation | $1358 | - Use ambulance fees as proxy for police costs – as no information on costs publicly available. |
| Police and ambulance required for transportation | $2716 | - Combine fees for ambulance and police as proxy for this cost, as no publicly available information |
| ED presentation –  not admitted | $552 | - Cost $472 sourced from Round 22 NHCDC data for Victoria 2017-18 - Uplifted for inflation using Reserve Bank of Australia inflation calculator (inflation 17% over 5 years) - 2022-23 cost $552.30 |
| ED presentation –  admitted | $1129 | - Cost $965 sourced from Round 22 NHCDC data for Victoria 2017-18 - Uplifted for inflation using Reserve Bank of Australia inflation calculator (inflation 17% over 5 years) - 2022-23 cost $1,129.16 |
| Family/friend accompanied person | $170 | - Based upon data collected from seven participants who completed Time 1, Time 2 and Time 3 economic evaluation questionnaires. Then extrapolated across all 94 patients admitted to the program - Average time in ED assumed to be 4 hours   - 61% of people are in ED for less than 4 hours and 90% less than 9 hours 15 mins as per AIHW statistics:   - Source: https://www.aihw.gov.au/reports-data/myhospitals/sectors/emergency-department-care - Average earnings per hour $42.50   - Source ABS – latest information 2021   - <https://www.abs.gov.au/statistics/labour/earnings-and-working-conditions/employee-earnings-and-hours-australia/latest-release> - Time valued using the opportunity cost approach   - Assumes value of the individual’s time equivalent to the average wage, regardless of whether the person was employed or not   - References: Trammer, Guerriere, Ungar, & Coyte, 2005; Zhang, Bansback, & Anis, 2011. - Cost of parent/friend accompanying young person $170 |
| Hospital stays per night | $1,261 | - As per AIHW mental health expenditure 2020-21 - <https://www.aihw.gov.au/mental-health/topic-areas/expenditure#public> - Used data for Victoria all services – included both acute and non-acute average, and all services such as child/adolescent, older adults and forensic. Child and adolescent highest cost hence use of general to cover those who may be adolescents in our sample. |
| QALY | $15,839 | - Valued benefit per QALY (Quality Adjusted Life Year) - QALY value source   - Pennington et al. (2015) in a large European study found individuals valued a QALY composed of QoL improvements at $US11,000.   - Higher values were for life extending gains (US19,000 avoiding coma, US$29,000 imminent, premature death)   - Surveys to inform QALY cost were conducted in 2010 - QALY calculation   - Converted to AUD$ as at 2010 at rate of US$ .92 ($US11,000/0.92 = AUD $11,957)   - Uplifted for inflation since 2010 using Reserve Bank of Australia inflation calculator AUD$ 11,957 = $15,839 per QALY |
