# Supplementary material for: Evaluation of a service introduced to support young people at risk of suicide using a cohort design, a mixed methods analysis and cost-benefit analysis
Source: PLOS Ment Health. 2024 Jun 10;1(1):e0000043. doi: 10.1371/journal.pmen.0000043 (PMC12798205; doi:10.1371/journal.pmen.0000043)
Supplement: Attachment — Submitted filename: response to reviewers.docx [file pmen.0000043.s002.docx]

Response to Reviewers

We are very grateful for the excellent advice you have provided in your review. Below we have explained how each of your suggestions have been addressed. Thank you very much indeed.

| **Reviewer Comment** | **Our response** |
| --- | --- |
| 1. The [PLOS Data policy](https://urldefense.com/v3/__https:/journals.plos.org/mentalhealth/s/data-availability__;!!NDYExDT0u85SdT4!tUCPoz5b0BE0DpyE-HZjZ9QB3_yTXtk4y9Db0GkjiMjkXzlVke4_NDbuoSvwJR8c2VFgPHCtrHm5d796IE_5$) requires authors to make all data underlying the findings described in their manuscript fully available without restriction, with rare exception. The data should be provided as part of the manuscript or its supporting information, or deposited to a public repository. For example, in addition to summary statistics, the data points behind means, medians and variance measures should be available. If there are restrictions on publicly sharing data—e.g. participant privacy or use of data from a third party—those must be specified. | Privacy concerns mean that we can only make restricted anonymised data available on request. Participants agreed to this study but not for the data to be made widely available in a non-aggregate manner. This was also stipulated in the ethics clearance for this project. This information has now been provided. |
| 2. Thank you for the opportunity to review the manuscript titled: 'Evaluation of a service introduced to support young people at risk of suicide using a cohort design, a mixed methods analysis and cost-benefit analysis'. The paper reports results of a worthy area of research and evaluation, that of preventing suicides in young people. I have made the following comments which I hope are helpful. | Thank you very much for your endorsement of our paper as a worthy area of research and evaluation. Your comments have all been most helpful. |
| **Abstract** |  |
| 3. The abstract would benefit from being clear regarding the dates of study and sample sizes obtained for the qualitative and quantitative data collection elements. It is also unclear whether the quantitative element is secondary data analysis or primary data collection; please can this be made more explicit. | Thank you. Added line 42  The evaluation involved a mixed methods analysis of data collected during the first 20 months of operation from February 2022 to September 2023. Ninety one young people were admitted to the service during this period, of whom 28 agreed to participate in the evaluation. Primary data were collected by telephone or online, and secondary data for hospital service use was collected for 70 of the young people. |
| **Introduction** |  |
| 4. Para 1: context is provided by %s of young people dying by suicide, but what is the n associated? This will help to provide a clearer context. | Thank you. Now added line 64  64 deaths by suicide represented 30.9% of all deaths in young people aged 15-17 and 304 deaths by suicide represented 32.4% of all deaths in those aged 18-24 years |
| 5. Thank you for clear context and rationale for the study on Pages 5-6, this makes it clear for the reader. | Thank you so much for this positive comment. |
| 6. Page 6 lines 108-110 can you reiterate which service, where that is based, and reach of the service. Also, it states here that this is an evaluation. Immediately following it states it's a co-designed service. Does the evaluation evaluate the co-design process, the service, or both? Please can this be made clearer. If it does not include an evaluation of the co-design process then there appears to be a lot of detail provided on the co-design aspect and challenges and not a lot of detail and context on the actual service, which is probably more useful and should be inserted if the evaluation focused on the service itself. | Thank you. We have reported more details on the service and we have replaced the detailed commentary on the co-design process with a single sentence. See line 110  The purpose of this study is to evaluate a new service to which children and young people under the age of 26 are referred after a suicide attempt, self-harm or persistent suicidal ideation. This service is being delivered within an existing public child and youth mental health service in Victoria, Australia. It encompasses time-limited support (maximum 3 months engagement) for young people aged under 25 years with an acute suicidal risk (recent attempt or persistent ideation), provided by a multidisciplinary team including psychiatric, psychological, psychosocial and lived experience staff. The team provides intensive, outreach wrap around support to the young person as well as their families when appropriate, includes liaison with other supports such as school wellbeing services, and concludes with a warm handover to ongoing supports where needed.     As recommended by the Royal Commission into Victoria’s Mental Health System, a co-design process was used to design this service, in order to acquire a comprehensive understanding of user needs and a greater diversity of ideas for meeting these needs [16].  As reported elsewhere co-design processes often pose implementation challenges, and the same was true for this program [17, 18, 19]. |
| 7. It is unclear what the outcome measures were for the evaluation; clarity on these would be needed in order to understand the results of the evaluation. | Thank you. Included line 140  This evaluation has been specifically designed to determine if there was a reduction in suicidal ideation and improvements in coping, self-efficacy, resilience and well-being for the young people after engaging with the service, and if this was sustained three to six months following discharge, resulting in reductions in the usage of hospital services. In addition, the objective was to obtain feedback from the young people themselves regarding their experience of the service and to conduct a cost-benefit analysis for the service. |
| **Methods** |  |
| 8. It is stated "However, the recruitment of young people for this evaluation study began on 5th April 2022" - can you make it clearer what the time period for the data analysed was. It implies no data before April, but can this be made clearer. | Thank you.  Reworded line 143  However, the recruitment of the 28 young people for the collection of primary data for this evaluation study began on 5^th^ April 2022, allowing time for the service to establish itself. Primary data collection ended at the end of September 2023. |
| 9. I assume, but this could be made clearer, that all young people who came through the service were approached to take part in an interview? Or was there a sampling frame? If yes, what? Please make this clearer. | Thank you. The following sentence has been added. Line 168  Apart from a few young people under the age of 12 or young people who were judged by the clinical treating team to be too ill, all the young people who came through the service during this period were invited to participate in the evaluation. |
| 10. Also see comment in abstract about quantitative methods being unclear. This is the same on Page 8 lines 164-175. Please can the methods be made much clearer. | Added line 149  Quantitative and qualitative data were collected in either telephone interviews or via online surveys that could be completed at a time and day that suited them. Mode was at participant preference. Further qualitative data were also collected from three telephone interviews.  Also please see 13 below.  Informative headings have been added throughout. |
| 11. Page 9 details Hospital Service Measures and others. Lines 178-183 in particular implies that the data collection period was different to the period April to Sept as stated earlier. Please can all data collection measures, methods, and data time points be made much clearer. This section also suggests a component of secondary data analysis and ties in with other comments around a lack of clarity on the methods used and data collection approaches. | Thank you. Added at line 137  All primary and secondary data collection was conducted in Melbourne, Australia, with the secondary data consisting of routinely collected hospital service data. A quantitative analysis including an economic evaluation was conducted using data from both these data sources, while a qualitative data analysis was conducted using only primary data.  More details provided under the headings Primary and Secondary Data Collection |
| 12. Re the measures, a lot of scales were used. Was there any feedback or thought given to participant burden? How long did it take for these to be completed by participants? | Thank you  Added line 191  *Service Evaluation Measures (Primary Data)*  A broad range of measures, partly informed by the prior evaluation of results for the similar adult service, were chosen in order to capture the varied and complex presentations expected. The constructs measured included suicidality, hope, quality of life, coping self-efficacy resilience and well-being. Advice from two psychologists within the service was sought regarding potential participant burden. Subsequent RedCap data indicated that it generally took 15-16 mins (with a range of 5 to 23 mins) for the survey to be completed online. However, it took up to 40 mins for the survey to be completed over the telephone.  These measures, described below, were collected at entry to the service, at discharge and at 3-months and 6-months follow-up using summated scales. |
| **Analysis** |  |
| 13. Page 13, lines 289-303 is not about analysis. This section needs moving to earlier in the methods as it's more about the design of the data collection instrument and not the analysis. It does also remind me that the methods are very sparse and means it would be infeasible for someone to fully know what was done, when, how and why. I would recommend revisiting Methods in terms of sampling, recruitment and data collection procedures and to make it clear for all components of the research. | Thank you. These lines have been moved.  Added line 248  In summary, young people over 17 completed seven scales (AHS, ORS, CSE, EQ-5D, PHQ4, SIDAS, RS-14), and young people under 17 completed six scales (CHE, ORS, CSE, EQ-5D, PHQ-A, CYRM-R). In addition, in the discharge and follow-up surveys, all the young people completed purposely designed questions specific to the evaluation in order to better understand the service experiences of the young people. For example, the young people were asked to rate the helpfulness of the service and the service team on a 4-point Likert scale.  The discharge and follow-up online surveys also included a variety of open-ended questions that allowed the collection of qualitative data. These were questions about what was found most helpful about the service and suggestions for improvement. The same questions were used for the three follow-up one-on-one telephone interviews.  **Secondary Data Collection**  Hospital service data were collected for HOPE service users for whom these data were available (N=70). These data were used in the quantitative analysis, including part of a cost-benefit analysis for the new service. Hospital service data were collected for the three months before admission to the service, during admission, and for the three and six months post admission, to establish to what extent hospital service use declined during and following service entry. These data considered a period of 3 months prior to service acceptance and up to 9 months after service acceptance, therefore covering the period from November 2021 to 30^th^ September 2023, when all data collection ceased.  Changes in Crisis Assessment Treatment Team (CATT) calls, Emergency Department (ED) presentations, hospital admissions and nights in hospital were collected for the young people aged 17 or over (*N*=70) and extrapolated over the 92 admissions to the HOPE service. It was not possible to obtain hospital service data for these participants. Twenty were under the age of 17 and attending specialist children services, and two were referred from a different hospital network. |
| **Results** |  |
| 14. This "Of these young people sixteen were declined service for various reasons" does not make sense and needs re-wording. | Thank you. Reworded line 343  However, 16 young people were deemed ineligible for service for various reasons (for example, not in catchment), leaving 91. |
| 15. "One young people" should be 'One young person'. | Apologies for this embarrassing error. Line 345 |
| 16. Page 15 Line 323 all acronyms here and elsewhere need defining in full first time stated. | Thank you. Done for CATT and ED. |
| 17. Page 20 states "There were several open-ended questions in the discharge and follow-up surveys that allowed the voices of the young people to resonate with their closed question responses." No where earlier in the methods information does it say qualitative data included open responses from a survey. Please ensure methods, including analysis, are clear throughout. | Thank you. Added under Primary Data Collection line 255  The discharge and follow-up online surveys also included a variety of open-ended questions that allowed the collection of qualitative data. These were questions about what was found most helpful about the service and suggestions for improvement. The same questions were used for the three follow-up one-on-one telephone interviews.  Added under Qualitative Data Analysis line 329  The open-ended questions in the discharge and follow-up online surveys and the three follow-up one-on-one telephone interviews were analysed using Braun and Clarke’s 6-stage thematic analysis approach [37].  Under Qualitative Results line 444  Twenty-six young people provided qualitative feedback in relation to the service, verbally or in written form. This feedback was collected through a combination of interview and open-text survey data. In particular, there was feedback about what they found most helpful about the service and what could be improved.  Under limitations added the following line 741  Our qualitative analysis also has limitations in that we relied mainly on online surveys for the collection of these data, with only three one-on-one telephone interviews conducted. Although online surveys are acceptable for the collection of qualitative data, this tends to result in less rich data. It also means that there is no opportunity to ask follow-up questions or prompt someone to elaborate. |
| 18. Are the interview themes a combination of interview and open-text survey data or just interview data? Please make clear. | Added under Qualitative Results.  Please see point 17. |
| 19. It is somewhat confusing having some interview quotations in tables and some in text. Why is this? Can it be made consistent? There is also very limited narrative about themes and sub-themes. I appreciate word counts of the journal but it almost comes across as qualitative findings/narrative is somewhat limited/rudimentary. | Thank you. This section has been rewritten for consistency. The tables have been removed. Narrative about themes added line 583 While these last suggestions are common to time-limited support services, the overall qualitative data indicates that the model of care which was chosen for the implementation of this new service is successful in meeting the needs of the cohort of young people who are presenting for care. In particular, the move away from traditional, medically or psychologically centred care offered in a clinical setting was greatly appreciated, allowing the young person to feel supported and held in their usual environment. Support from workers with Lived Experience for both the young person and their parent, as well as other forms of psychosocial and community-based support was welcomed, as it helped the young person and family feel that they were able to get on with their lives. While the service also provides clinical support through traditional models such as medication reviews and access to psychological therapies, young people were less likely to mention these aspects of the service as ones which stood out as being especially helpful. This may be because they more closely conform to what the young person may have expected to receive, and were thus less likely to arouse comment. |
| 20. It is hard to contextualise the qualitative responses/findings because there is very limited context provided earlier in the paper on the service, it's components etc. | Description of service added. Please see Comment 6. |
| **Discussion** |  |
| 21. I think this sentence is somewhat far-reaching given the results earlier presented "In addition, significant improvements in quality of life have been observed and a cost-benefit analysis has indicated that the service is also likely to be economically justified if these benefits can be retained over more than a 12 month period.". | Thank you. This claim has been made more accurate. Line 604    In addition, significant improvements in quality of life have been observed and a cost-benefit analysis has suggested that the service may be economically justified if these benefits can be retained over more than a 12 month period. |
| 22. Is there any evaluation data that could suggest reasons for attrition? Presumably this is also linked to their current mental condition and mental capacity to participate? Any data/findings to comment on here? | Thank you. Possible reasons for attrition include   a. An over burdensome questionnaire b. Work pressures as suggested by higher attrition rates in older young people. c. Success of the service means people are moving on with their lives – may not have time/ interest in revisiting a period of their lives that was unhappy     Added under Limitations line 723    The age bias shown in the attrition rate may be due to employment pressures, also perhaps pointing to an over-burdensome survey questionnaire, but needs further investigation. It is also possible that the young people prefer to move directly back to the life they had previously, without the need to reflect on their recovery journey. |
| 23. It is hard to contextualise the discussion because there is very limited context provided earlier in the paper on the service, it's components etc. It is difficult to see which service elements are well thought of or not and how widespread or not both positive and negative views were. | Description of the service added as per Comment 6. |
| **Implications** |  |
| 24. Implications seem to centre on co-design challenges, yet no results are actually presented which touch on co-design. Please ensure implications closely follow findings reported in this paper and not elsewhere. | Thank you. Implications section has been rewritten with the following added line 701    In addition, an integrated and coordinated service system, which catches young people at critical junctures in their mental healthcare journey, such as at discharge from an inpatient unit, will be better placed to stop young people falling through the cracks. Having a service which builds-in referral and handover to ongoing care, helps to develop a better integrated system across hospitals, community care, schools and other services.  The evaluation team experienced difficulties in recruiting young people for this study. Traditional research methods for contacting the young people were often not successful, suggesting that alternative approaches, perhaps via social media platforms that young people frequent, would have been more effective. Closer adherence to the ideals of co-evaluation, with the inclusion of a young person with lived experience of suicide on the evaluation team, is therefore recommended for future studies. |
